# A Systematic Review and Meta-Analysis of the Relationship Between Brain Data and the Outcome in Disorders of Consciousness

**DOI:** 10.3389/fneur.2018.00315

**Published:** 2018-05-08

**Authors:** Boris Kotchoubey, Yuri G. Pavlov

**Affiliations:** ^1^Institute of Medical Psychology, University of Tübingen, Tübingen, Germany; ^2^Department of Psychology, Ural Federal University, Yekaterinburg, Russia

**Keywords:** consciousness, improvement criteria, meta-analysis, minimally conscious state, neurophysiological markers, prognosis, publication bias, unresponsive wakefulness syndrome

## Abstract

A systematic search revealed 68 empirical studies of neurophysiological [EEG, event-related brain potential (ERP), fMRI, PET] variables as potential outcome predictors in patients with Disorders of Consciousness (diagnoses Unresponsive Wakefulness Syndrome [UWS] and Minimally Conscious State [MCS]). Data of 47 publications could be presented in a quantitative manner and systematically reviewed. Insufficient power and the lack of an appropriate description of patient selection each characterized about a half of all publications. In more than 80% studies, neurologists who evaluated the patients’ outcomes were familiar with the results of neurophysiological tests conducted before, and may, therefore, have been influenced by this knowledge. In most subsamples of datasets, effect size significantly correlated with its standard error, indicating publication bias toward positive results. Neurophysiological data predicted the transition from UWS to MCS substantially better than they predicted the recovery of consciousness (i.e., the transition from UWS or MCS to exit-MCS). A meta-analysis was carried out for predictor groups including at least three independent studies with N > 10 per predictor per improvement criterion (i.e., transition to MCS versus recovery). Oscillatory EEG responses were the only predictor group whose effect attained significance for both improvement criteria. Other perspective variables, whose true prognostic value should be explored in future studies, are sleep spindles in the EEG and the somatosensory cortical response N20. Contrary to what could be expected on the basis of neuroscience theory, the poorest prognostic effects were shown for fMRI responses to stimulation and for the ERP component P300. The meta-analytic results should be regarded as preliminary given the presence of numerous biases in the data.

…the quality of methodological reporting in the social and behavioral science research literature is poor. Reports are often silent or ambiguous on important methodological and procedural matters making it difficult for the analyst to determine what was done. The metaanalyst who develops elaborate and detailed methodological criteria for study selection, therefore, will most likely find that study reports do not provide sufficient information for those criteria to be confidently applied. [Lipsey and Wilson (1) (p. 22)]

A systematic analysis of several published datasets can yield substantial new knowledge as compared with the data of each single experiment (2). This insight, increasingly admitted during the last decades, underlies the use of meta-analyses and other kinds of quantitative reviews that can largely overcome the subjectivity and deliberateness of the “good old” narrative reviews. The domain of the severe Disorders of Consciousness (DoC) is, however, still dominated by the latter genre. Thus a brief overview of journal publications about brain imaging data in DoC for the last 5 years reveals that almost every fourth paper (exactly, 33 of the 137 papers) is a narrative review. Book publications further increase this number.

The notion DoC most usually includes two diagnostic entities: the vegetative state, or unresponsive wakefulness syndrome (UWS), and the minimally conscious state (MCS) (3, 4). To the best of our knowledge, the first systematic analysis of neurophysiological data in DoC was devoted to the question whether these data confirm the reality of the distinction between UWS and MCS (5). The authors came to the conclusion that there were no reliable differences in terms of neurophysiological variables (mainly EEG, PET, and fMRI) between the two diagnoses.

Hannawi et al. (6) concentrated on brain imaging studies and performed a voxel-based meta-analysis of 13 PET and fMRI studies in DoC patients, in which the corresponding data were reported. The authors identified a number of structures whose resting state activity was significantly decreased in patients as compared with healthy controls. On the other hand, they did not find convincing differences between UWS and MCS, which was in line with Liberati et al. (5). Kondziella et al. (7) came, however, to a different conclusion that brain connectivity data in rest and under passive stimulation (but not in active instruction conditions) reliably differ between UWS and MCS. Unfortunately, inclusion criteria in this study were not completely clear; thus the question still remains open whether late event-related brain potential (ERP) components (P300, N400) can be regarded as indicators of cortical connectivity, and therefore, the authors should either include all P300 and N400 studies in their analysis (if they answer this question positively), or exclude all of them (if they answer it negatively), but instead, they included only some of them. The data were not checked for publication bias, that is, the tendency for positive results or stronger effects to get published more readily than negative results or weaker effects (8). The simplest index of this bias is a negative correlation between the size of the obtained effect and its reliability (9). On the other hand, Kondziella et al. (7) indicated a bias in patient selection. The risk of this bias was estimated as “high” in 81.4% of the analyzed studies and as “uncertain” in further 11.6%.

Also, Bender et al. (10) were interested in the abilities of neurophysiological techniques to distinguish between UWS and MCS. Their meta-analysis aimed not at the presence and size of the effects, but at the parameters of sensitivity and specificity. The authors concluded “… that modern diagnostic techniques can already make a major contribution to the diagnostic assessment of MCS.” The inspection of their empirical findings yields a modest support for this conclusion, because good sensitivity and specificity values were found only for the measures of quantitative EEG; ERP and fMRI measures revealed, to the contrary, only moderate specificity and rather low sensitivity that did not significantly differ from chance.

Kotchoubey (11) carried out a quantitative analysis of 61 reports on ERPs in DoC. ERPs are the most frequently used neurophysiological technique in DoC, which, however, does not mean that they are also most useful. In general, the results of the analysis were rather disappointing. Most studies possessed such a low statistical power that their findings can at best be regarded as “preliminary results.” In addition, there was strong evidence for a publication bias toward positive findings.

However, there were good news. The above-mentioned deficits mainly concerned the studies where ERPs were compared between UWS and MCS, which largely concurs with the conclusions of Liberati et al. (5). The negative tendencies were substantially less expressed in the literature about the relationship between ERP and the prognosis of DoC outcome. Furthermore, the power of the prognostic studies correlated positively, and the effect sizes (ESs) correlated negatively, with the rank of journals where the data were reported. This indicated that weaker but more reliable effects could be published more successfully in top-ranking journals than strong but less reliable ones.

Like all areas in which there is no golden diagnostic standard, meta-analyses of novel diagnostic tests in the domain of DoC have a strong circular component. The expensive neurophysiological techniques are developed to complement imperfect clinical methods and to increase the diagnostic precision; but in a meta-analysis, these novel techniques are evaluated on the basis of the same (presumably imprecise) diagnostic criteria that these methods should improve! The lack of the golden diagnostic standard makes another strategy more preferable, i.e., a search for the measurements most reliably related to prognosis. The above-cited findings, that prognostic studies in DoC appear to have a higher quality than diagnostic studies, are in line with this view.

The aim of the present study was a systematic analysis of all publications relating any functional brain data recorded in UWS and MCS patients to their outcome several weeks or months after the measurement. Data using only anatomical brain measurements were not included in the present analysis. Each of the analyzed publications will hereafter be designated as “record.” The term “dataset” will, in contrast, refer to any individual comparison between a neurophysiological variable (e.g., fMRI activation in a specific task) and an outcome variable (e.g., Glasgow Outcome Scale—Extended, or GOSE (12)). One record can, therefore, contain many datasets.

## Methods

### Literature Search and ES Calculation

**Figure 1:**
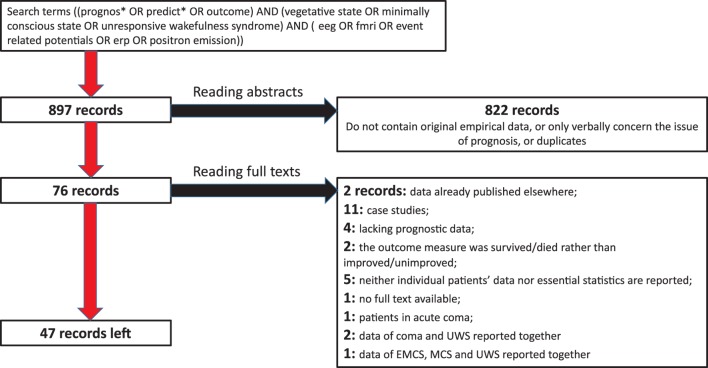
Flow chart of the selection of records.

A search in MEDLINE and SCOPUS was conducted on the 23 November 2017 by using search terms ((prognos* OR predict* OR outcome) AND (vegetative state OR minimally conscious state OR unresponsive wakefulness syndrome)) AND (eeg OR fmri OR event related potentials OR erp OR positron emission). No time limits were set for the search. In addition, the systematic reviews cited above in the Introduction as well as recent informal reviews on neurophysiology of DoC, were consulted. Eight hundred ninety-seven peer-reviewed records published in English, German, or Russian, were identified. After reading abstracts and removing duplicates, 822 of them were rejected as irrelevant. Full test was sought for the other 76 records. Seven of them were rejected because they did not contain any outcome data on DoC patients, or contained outcome data already published elsewhere. Further exclusion criteria were (a) case studies or series of cases; (b) using patients’ survival, and not the clinical improvement, as the only prognostic criterion; (c) presentation of the results in such a general form that the size of the observed effects cannot be calculated; and (d) reporting the data of UWS and MCS patients together with other diagnoses such as coma, exit-MCS, or locked-in syndrome: on the basis of these criteria 22 records were rejected. Regarding (d), we accepted studies in which UWS and MCS data were reported together, but not those in which the sample included more than these two diagnoses and the reported data did not give the reader a possibility to distinguish between the different diagnostic groups. The process of the selection of relevant records is shown in Figure 1.

The search resulted in 47 records containing a total of 381 datasets. These records are summarized in Table 1. Effect size was calculated for each dataset (i.e., for each predictor–outcome pair) on the basis of primary data on each individual patient presented in the tables of most studies, or chi-squared based on the same patient data, or the *t*-statistics. Only in one record, ES was calculated from a coefficient of correlation. All these parameters were converted into Cohen’s *d* following the methods summarized by Lenhard and Lenhard (13). If a resulting 2 × 2 table contained a zero cell (e.g., all patients having a positive neurophysiological sign recovered), the blind application of the corresponding formulas results in *d* = infinite; to avoid this, we added 0.5 to all cells, as recommended by Nakagawa and Cuthill (14). When *d*-values were included in further operations (added, averaged, etc.), they were weighted by inverse standard error (SE).

**Table 1:** A summary of the 47 records included in the systematic review.

Reference	*N* (UWS/MCS)	Follow up, months	Methods	Relevant measures	Improvement criteria^a^
(15)	64/0	2	EEG, 24-h polysomnography	General sleep patterns Dominant EEG rhythm: beta, theta or delta	Diagnosis MCS, or GOS > 2
(16)	14/4	6–38	24-h polysomnography	Sleep complexity, presence of different sleep stages	CRS-R
(17)	10/0	36	~14-h polysomnography	Sleep complexity, number of sleep spindles, presence of different sleep stages	GOS
(18)	42/0	3	EEG	Spectral power of resting state EEG in delta, theta, alpha1, alpha2, beta1 and beta2 frequency bands	LCF > 5
(19)	59/47	3	EEG	EEG amplitude normality (>20 μV), dominant frequency, reactivity to forced eyes opening EEG combined AFR index	CRS-R; change from UWS to MCS or from MCS to EMCS
(20)	28/0	6	EEG	The same as in Ref. (19)	CRS-R; change from UWS to MCS or from MCS to EMCS
(21)	12/1	3	EEG	EEG normality according to Synek scale	LCF
(22)	4/5	6	Auditory ERP	MMN, N2 and/or P300 in control and after listening to music condition	CRS-R ≥ MCS
(23)	34/0	24	Short-latency EP, somatosensory EP, EEG	BAEPs grade, EEG reactivity to passive eyes opening, pain and acoustic stimuli, EEG Synek index, N20 SEP grade, P300 to a patient’s own name	DRS < 22
(24)	17	12	PET	FDG-PET in the resting state	GOSE > 2
(25)	7/0	2–9	fMRI	BOLD response to speech and noise stimulation	CRS-R ≥ MCS
(26)	22/16	6	fMRI	fMRI BOLD response to speech and sound stimulation	CRS-R
(27)	7/4	3	fMRI	fMRI BOLD response to subject’s own name	CRS = MCS
(28)	3/7	6	EEG, fMRI	EEG, BOLD response to language, music, active motor imagery instruction	GOSE > 2
(29)	43/0	24	EEG, somatosensory EP	EEG classified according to Synek scale N20 SEP grade	CRS-R ≥ MCS
(30)	14/0	3	EEG	Resting state EEG Index of Structural Synchrony (amplitude, length, instability, number of functional connections in Alpha, Beta1, Beta2 bands)	LCF = MCS
(31)	8/0	24	Auditory ERP	N2, P300	Recovery of awareness but no standardized assessment
(32)	20/0	NA	Auditory and visual ERP, SPECT	Auditory MMN, N100, N200, P300; visual EP present/absent; resting state brain metabolism assessed by SPECT	GOS > 2
(33)	75/38	4	Somatosensory EP	N20	CRS scores of ≥ 23
(34)	56/0	12	EEG, somatosensory EP, 24-h polysomnography	EEG reactivity to noxious stimulation, N20, sleep spindles in 24-h EEG All predictors scored as absent or present	GOS > 2 or transition UWS to MCS
(35)	10/0	3	Somatosensory EP	N20 grade and latency	Recovery of awareness but no standardized assessment
(36)	1/4	3	fMRI	fMRI default mode network normality	Level of consciousness according to the Multi-Society Task Force on PVS
(37, 38)	24/19	6	Auditory ERP, EEG	MMN, N400, EEG dominant background activity	DRS ≥ MCS
(39)	6/5	12	EEG, fMRI	EEG reactivity to warm water stimulation, fMRI activation to thermal stimulation	GOS > 2 or transition from UWS to MCS
(40)	12/10	1, 2, 3, 6, 9, 12	Auditory ERP	P300	CRS-R ≥ MCS
(41)	50/0	5	EEG	EEG normality according to Synek scale, EEG reactivity to pain stimulation	Regaining consciousness according to GOS, LCF
(42)	23/0	6	fMRI	fMRI BOLD response to speech (adapted affective speech)	GOS > 2 or transition from UWS to MCS
(43)	11/0	6	Polysomnography	REM sleep characteristics	Recovery of awareness but no standardized assessment
(44)	6/2	3	Auditory ERP	MMN to subject’s own name stimuli, N100	CRS-R
(45)	6/5	3	PET	PET global GABA A receptor binding	CRS-R
(46)	52/0	3	fMRI	fMRI resting state connectivity	GOS > 2
(47)	5/0	0.5–2	EEG-TMS	TMS-evoked cortical responses	CRS-R
(48)	38/0	6	EEG	Resting state EEG Approximate Entropy, EEG reactivity (stimulation protocol is not described)	GOSE > 2
(49)	56/0	12	EEG	Spectral power in Delta, Alpha, Theta, Beta, Gamma frequency bands	CRS-R = MCS
(50)	71/0	1.5	EEG, auditory ERP	92 measures including: CNV, MMN, P1, P3a, P3b; normalized and absolute spectral power of delta, theta, alpha, beta, gamma rhythms; permutation entropy, Komolgorov–Chaitin Complexity; phase lag index (PLI), spectral entropy, imaginary coherence and weighted symbolic mutual information (wSMI) in different frequency bands	CRS-R
(51)	18/51	12	PET, fMRI	Resting state FDG-PET, BOLD response to active motor and visuospatial imagery tasks	GOSE > 2
(52)	53/39	24	Auditory ERP	N400, P300	CRS-R = EMCS
(53)	9/0	2–54	PET	Resting state FDG-PET	Recovery of awareness but no standardized assessment
(54)	10/12	1–6	fMRI	BOLD response to active motor and visuospatial imagery tasks	CRS-R; change from UWS to MCS, or MCS to EMCS
(55)	39/25	12	fMRI	fMRI BOLD response to subject’s own name	CRS-R
(56)	6/5	6	Auditory ERP	MMN, P300 to subject’s own name	CRS-R
(57)	10/0	24	Auditory ERP	MMN	LoC > 6
(58)	10/0	24	Auditory ERP	N200, N350, P300 in active and passive paradigms	LoC > 6
(59)	11/0	26–36	Visual ERP	N2, N3, P2 amplitude and latency, P2–P3 peak to peak magnitudes of VEP	LoC > 6
(60)	10/8	1–150	EEG, 24-h polysomnography	Permutation entropy, alpha-to-theta ratio, density of slow waves, high-to-low frequencies ratio, density of sleep spindles	GOSE > 2 or CRS-R
(61)	21/0	6	Short-latency EP, EEG, somatosensory EP	BAEP, N20 SEP grade, EEG normality, approximate entropy (ApEn), cross-approximate entropy, Lempel–Ziv complexity to pain, auditory and music stimulation in comparison with eyes-closed condition	GOS > 2
(62)	36/0	12 (after injury)	Somatosensory ERP	N20, P25, N20–N25 SEP grade and amplitude	GCS ≥ MCS

*N (UWS/MCS) means the number of UWS and MCS patients whose outcome and neurophysiological data were available. In the case of different number of patients available for different neurophysiological measurements, only the largest number is reported; it may be less than the total number of patients in the study*.

*^a^If GOS(E), CRS-R, LCF mentioned with no additional description, it was possible to calculate improvement criteria either way*.

*NA, not available, AFR index, Amplitude/Frequency/Reactivity index; UWS, unresponsive wakefulness syndrome; MCS, minimally conscious state; EMCS, Exit form MCS; GOS(E), Glasgow Outcome Scale (Extended), GCS, Glasgow Coma Scale; DRS, Disability Rating Scale; LCF, levels of cognitive functioning scale; LoC, Level of Consciousness Scale; SEP, somatosensory evoked potentials; BAEP, brain stem auditory evoked potentials; dwPLI, debiased weighted phase lag index; SPECT, single-photon emission computed tomography; CRS-R, Coma Recovery Scale-Revised*.

A big and still underestimated problem of all quantitative reviews is the plenty of non-reported data. Several authors (63–65) indicated that measured but unreported variables constitute one of the main sources of false positive findings ubiquitous in biology and psychology and thus an important cause of the contemporary “replication crisis” (66). When, and only when, it was evident for both present authors from the text of a paper that a neurophysiological variable was measured but not reported in relation to the outcome (or, rarely, reported as “non-significant”), the ES of this variable was assumed to be 0, and the SE of ES was assumed to be equal to the median SE calculated for the reported variables in the same record.

When the results presented several strongly correlated predictor variables (e.g., the same EEG variable in several adjacent regions), they were regarded as representing the same “construct” (1), and the mean and standard deviation (SD) for the construct were calculated according to the formulas

$$\text{Mean}=\text{(}M_{1}+M_{2})/N;$$

$$\text{SD}=\sqrt{\frac{\left(N-1\right)\left({\text{SD}}_{1}^{2}+{\text{SD}}_{2}^{2}\right)+0.5N\left(M_{1}+M_{2}-2M_{1}M_{2}\right)}{2N-1}}$$

where *M*_1_ and *M*_2_, SD_1_ and SD_2_ are mean and SD values for two to-be-combined variables, respectively. (Note that the formulas are so simple because both variables have the same *N*.)

### Outcome Criteria

The category “bad outcome” for UWS and MCS patients presumed remaining in the same condition. Deaths were included in this category only if it was clear that a patient died as a direct consequence of the brain lesion, otherwise excluded from the analysis. The category “good outcome” has, on the other hand, two different definitions: minimal clinical improvement or regaining full consciousness.^1^ For MCS, both criteria are the same, because their minimal improvement implies the transition to Exit-MCS. But this is not true for UWS, because their minimal improvement means only the transition to MCS. As shown in Section “Results,” the two different improvement criteria of UWS yield different results.

Avantaggiato et al. (17) analyzed a group of DoC patients containing children and adolescents; because the authors presented individual data of each patient, we selected the results for patients >13 years only. The category “good prognosis” for MCS implied the recovery of consciousness. For UWS, however, it might include either the recovery of consciousness or the transition into MCS. All 381 datasets were included in the systematic review.

### Quality Assessment

Quality of the records was estimated on the basis of the QUADAS criteria (67) that have been tailored, as recommended in the original publication, for the specific research field. Because Kondziella et al. (7) expressed concerns about possible bias in patient selection, we recorded whether a publication included a patient flow chart, and whether it described explicit exclusion criteria for UWS and MCS patients or simply mentioned that all patients admitted in the clinics for a particular time period were investigated. We also marked the records in which obviously more neurophysiological data were collected than reported in the analysis of outcome prediction. Another quality index was the use of the Coma Recovery Scale-Revised (CRS-R (68)) for DoC diagnostics, because this scale, though not being golden standard, possesses substantially better psychometric qualities than all other instruments for the assessment of DoC (69). In addition, the impact factor (IF) of the publishing journal was included as an indirect quality criterion, because the data indicate high correlation between the IF and the informal reputation of the corresponding journal among neurologists (70).

### Meta-Analysis

Two additional inclusion criteria for meta-analyses were (1) at least three independent records reporting the same predictor variable or variables related to the same construct and (2) each of these records includes at least 10 patients. The criteria can be regarded as very liberal because, first, only three records and only 10 patients (who further should be subdivided into at least two groups) are rather low numbers, and second, the notion of construct is rather vague and permits to include into one group, for example, studies of P300 to simple tones and to patients’ own names, thus increasing heterogeneity. On the basis of these criteria, 319 datasets were excluded (of course, this number would be larger if the criteria were more conservative). The remaining 62 datasets finally entered the meta-analysis.

We used a random-effects meta-analysis with restricted maximum-likelihood (REML) estimator for pooling ESs. We assessed the level of heterogeneity between studies with a standard *Q*-test statistic as well as by *I*^2^ calculation (71). Heterogeneity was regarded as significant when *p* < 0.05 or *I*^2^ > 50%. Potential publication bias for individual predictors was assessed with the Egger test for Funnel plot asymmetry and represented graphically with Begg’s funnel plots of the ES versus its SE. Additionally, Rosenthal fail-safe test was also applied. All meta-analyses were performed using R package “metafor” (72) using inverted standard errors as weighting parameter.

## Results

### Quality of Reporting

None of the 47 records presented a flow chart depicting patient selection. The authors of 20 records (42.6%) explicitly state that they included all patients within some exactly described time period. Nine reports (19.1%) depicted at least some inclusion and/or exclusion criteria. In the remaining 18 records, patient selection was not described.

Most selected records present their data either for each individual patient or as mean ± SD (or SE) for each relevant group (e.g., recovered versus non-recovered). Three records present data only in a general form (e.g., as correlations). Five records (10.6%) mention the size of some effects.

CRS-R was used for the diagnosis of UWS and MCS in 29 records (61.7%); other studies employed Disability Rating Scale, Glasgow Coma Scale, or other less powerful instruments.

Quite surprisingly, only two records explicitly state that the neurologists who assessed the outcome were blinded to the neurophysiological data collected before. In six records, blindness of the outcome might be assumed because neurophysiological examination and outcome diagnostics were performed in different institutions. These eight records (17%) were combined into one “blind” group. In the majority of the records (83%), the outcome was diagnosed with knowledge of the neurophysiological findings.

The median time between the measurement and the outcome assessment was 6 months, mean minimal time per record was 9 months (range 1–36 months), and the mean maximal time per record 16 months (range 1.5–150 months). In 42 records (89%), this interval was same for all the examined patients. Seven records used broad variable intervals for different patients (1–6, 1–30, 2–9, 6–38, 10–150, 26–36, and 24–144 months). One publication does not report the measurement–outcome interval.

The mean total sample was 31.11 ± 3.66 patients, with a median of 21 patients and a range of 5–123 patients. Eight records included <10 patients, 14 records had between 10 and 19 patients. Adding the case studies filtered out at the previous stage, we come to the result that about a half of all prognostic studies included <20 patients. The records that did not describe patient selection included significantly less patients (means 14.1 versus 43.0, *t* = 5.11, *p* < 0.001) than records describing their selection process.

The median IF of the publishing journals was 3.87, range from 0 to 44. IF did not differ between the records with correctly versus incorrectly described patient selection. We hypothesized that studies with more patients (thus having higher power) are published in more prestigious journals, but the corresponding correlation was not significant (Spearman’s ρ = 0.26, *p* = 0.078). However, studies employing CRS-R were published in journals with higher IF than studies that did not use this scale: *p* < 0.001, Mann–Whitney test. A few studies with blind outcome assessment included larger sample sizes than studies without outcome blindness (*t* = 2.35, *p* = 0.023) and were also published in more prestigious journals (*p* = 0.046, Mann–Whitney test).

Notably, we did not find a significant relationship between any of the variables and the time elapsed from the neurophysiological measurement till the assessment of the outcome. Non-weighted ES correlated with the mean time between neurophysiological measurement and outcome assessment with Spearman’s ρ = −0.09, with minimal time per study ρ = −0.18, with maximal time per study ρ = −0.07 (all nonsignificant). For weighted ES, the corresponding correlations were 0.03, −0.06, and −0.11, respectively (all nonsignificant). Likewise, correlations of the time interval with the SD of ES (as a measure of its reliability) were all between 0.00 and 0.03, and correlations of the time interval with sample size were between 0.00 and 0.06, all non-significant.

Also, the year of publication did not correlate with any other measures. The bibliographic literature gives a reason to expect that later publications might have large samples or smaller ESs than earlier (73). Although the corresponding correlations were in the expected direction, they did not reach significance (year/N: Spearman’s ρ = 0.12; year/ES: ρ = −0.08). Also, the correlation between publication year and IF was close to 0 (ρ = 0.03).

### Publication Bias

**Figure 2:**
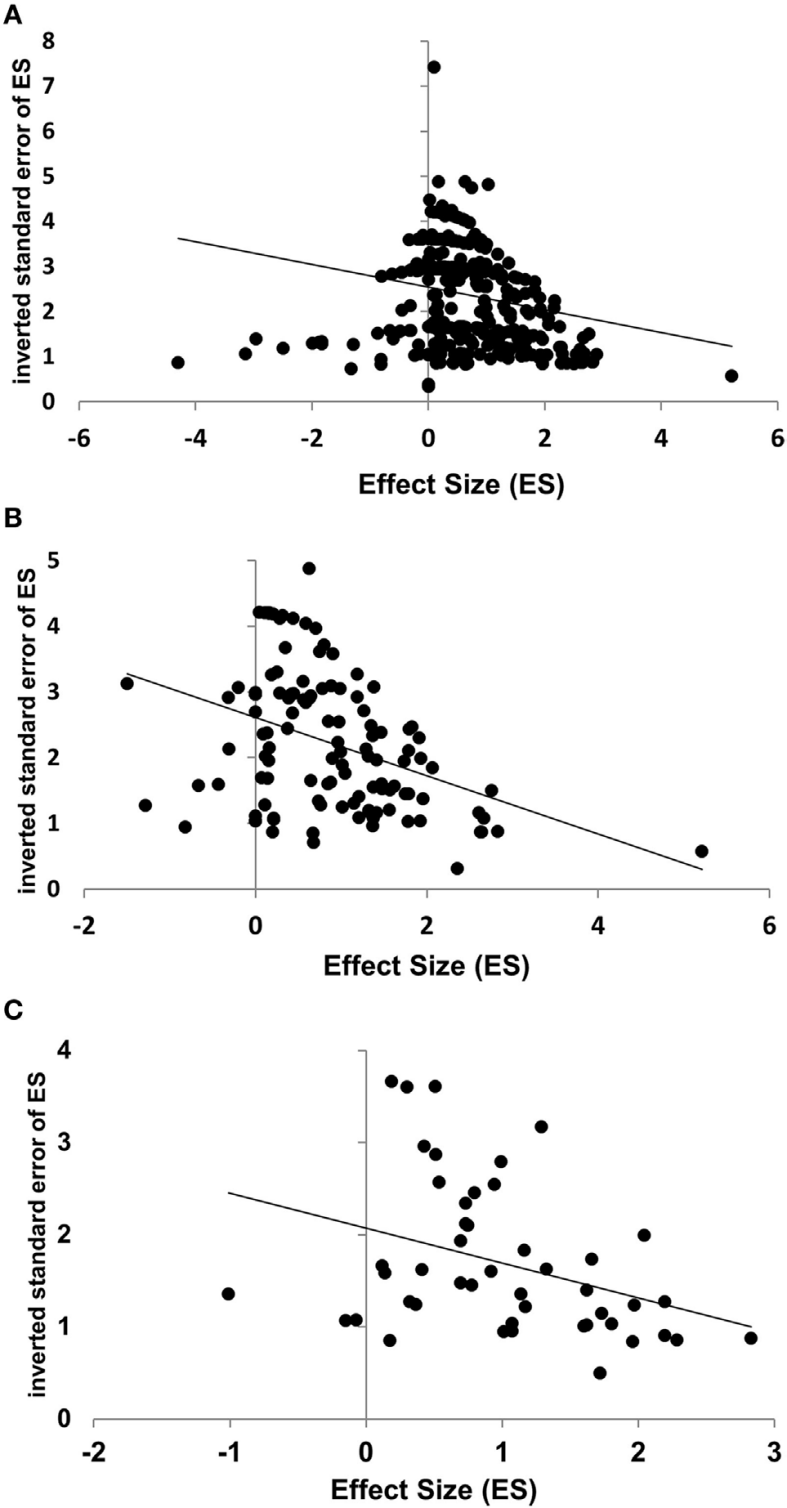
Negative correlations between effect size (ES) and its reliability, estimated by the inverted standard error (1/SE), for all individual datasets [**(A)**: Spearman’s ρ = −0.22, *p* < 0.001], datasets included in the meta-analysis [**(B)**: ρ = −0.47, *p* < 0.001], and for mean ESs per record [**(C)**: ρ = −0.41, *p* = 0.004]. The regression lines are presented for illustration only, not for quantitative analysis.

The inverted SE (1/SE) was taken as a measure of the reliability of an ES. Across all datasets, the rank-order correlation between ES and its 1/SE was weak but significant (ρ = −0.22, *k* = 381, *p* < 0.001). The result might be biased because different records contribute disproportionally to the whole mass of data. However, the positive correlation between the ES and its SE became even stronger when calculated for the selected subset of datasets included in the meta-analysis (ρ = −0.47, *k* = 62, *p* < 0.001), as well as for the ESs averaged for each record (ρ = −0.41, *k* = 47, *p* = 0.004). The results are shown in Figure 2.

Note that the first analysis (across all datasets) overestimates the contribution of the records reporting many datasets. The last analysis (across averaged ES), to the contrary, may overestimate the contribution of the records presenting few or only one dataset. Despite this contrary bias, very similar results were obtained. To sum up, these data show a trend to selective publication of strong but unreliable effects. How serious the bias is in respect of each particular predictor variable will be discussed below.

### UWS and MCS

Thirteen of the 381 datasets included only MCS samples, 248 datasets included only UWS patients, and the remaining datasets included both diagnostic groups of DoC patients.

**Figure 3:**
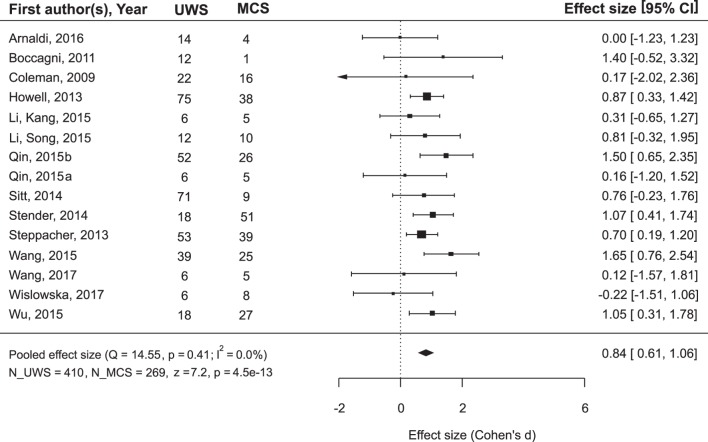
The results of the meta-analysis for prediction of the outcome from the diagnosis. The criterion of improvement for all patients was recovery of full consciousness. *Q*, the corresponding *p*-value and *I*^2^ are estimates of between-study heterogeneity; symbols ■ stay for the estimates of effect size (ES) in each single study, with the size of the symbol being proportional to the precision of the estimate. Error bars indicate the 95% confidence intervals of ES. The diamond ♦ is the estimate of the overall effect, the edges of the diamond represent the 95% confidence interval limits; CI, confidence interval; UWS and MCS, sample size of UWS and MCS patients in individual studies; N_UWS and N_MCS, overall sample size of the two patient groups. The resulting ES was tested for significance using *z*-criterion; the values of *z* and the corresponding *p* are given at the end of the lower left line.

**Figure 4:**
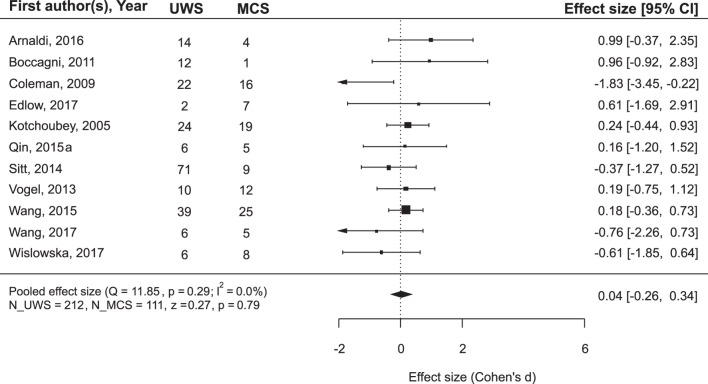
The results of the meta-analysis for prediction of the outcome from the diagnosis. The criterion of improvement was “minimal improvement,” that is for UWS patients, it was the transition to MCS, and for MCS patients, at least the transition to Exit-MCS. As can be seen, with this improvement criterion the diagnosis does not predict outcome. The rest is the same as in Figure 3.

While the main issue of the present study was outcome prediction on the basis of neurophysiological data, we also asked the question whether the outcome can be predicted simply from the diagnosis. Many authors of the reviewed articles also asked this question and answered it negatively. However, a meta-analysis of the combined data from the records where both diagnosis and prognosis could be followed revealed that MCS patients recovered consciousness significantly more frequently than UWS patients (Figure 3): mean ES = 0.84, 95% CI from 0.61 to 1.06. On the other hand, if the positive outcome of UWS patients is defined as any minimal improvement, i.e., the transition to the MCS, the diagnosis loses its predictory value (Figure 4).

Because we found that the improvement criterion for UWS (transition to the MCS versus recovery of full consciousness, that is, exit-MCS) can play a role in the calculation of prediction effects, we compared weighted mean ES for the neurophysiological variables in three conditions: (i) prediction of the recovery of consciousness for MCS patients; (ii) prediction of the recovery of consciousness for UWS patients; and (iii) prediction of the transition to MCS for UWS patients. A one-way ANOVA across these three groups resulted in a highly significant effect: *F*(2,294) = 23.11, *p* < 0.001. The result does not change when we limit the analysis by only those datasets that will later enter the meta-analysis [*F*(2,60) = 19.88, *p* < 0.001], or when we exclude all mixed datasets [*F*(2,86) = 12.08, *p* < 0.001]. Independently of the method of calculation, the mean weighted ES for the groups (i) and (ii) (i.e., different diagnoses, the same improvement criterion) were very similar and varied—dependent on the selected data—between 0.41 and 0.48. The mean weighted ES for the group (iii) was about three times larger (between 1.40 and 1.68) and differed significantly from both of them, although the groups (iii) and (ii) included patients with the same diagnosis and some datasets involved in these two groups might even include some of the same patients. To sum up, neurophysiological methods are significantly more successful in prediction of the transition from UWS to MCS than in prediction of the recovery of full consciousness.

### Meta-Analysis of Predictory Constructs

According to the above results, we performed the meta-analysis separately for (a) prediction of any clinical improvement (for which UWS patients means at least transition to the MCS), and (b) prediction of the recovery of full consciousness. Sixty-two datasets comprising a total of 1,919 patients were analyzed. They involved the following potential predictors:

1. EEG reactivity to “passive” stimulation (i.e., without an active instruction). Hypothesis: stronger EEG oscillatory responses = > better prognosis.
2. EEG entropy indices. Hypothesis: higher EEG entropy = > better prognosis.
3. EEG dominant oscillatory activity. Hypothesis: background activity closer to the alpha frequency = > better prognosis.
4. EEG Synek score (74) is frequently used in the intensive care medicine for the prognosis of the outcome of acute coma. Hypothesis: higher score = > better prognosis.
5. fMRI BOLD response to passive (auditory or nociceptive) stimulation. Hypothesis: stronger response = > better prognosis.
6. Resting state PET or SPECT metabolism. Hypothesis: closer to normal brain metabolism = > better prognosis.
7. N20 component of somatosensory evoked potentials (SSEP). Hypothesis: normal N20 = > better prognosis.
8. Auditory Mismatch Negativity (MMN) to a change in ongoing acoustic stimulation. Hypothesis: larger MMN = > better prognosis.
9. Auditory P300 as an index of complex processing in cortico-subcortical networks. Hypothesis: larger amplitude or shorter latency = > better prognosis.
10. Spindle activity as an index of information processing in sleep (75). Hypothesis: presence of sleep spindles = > better prognosis.

**Figure 5:**
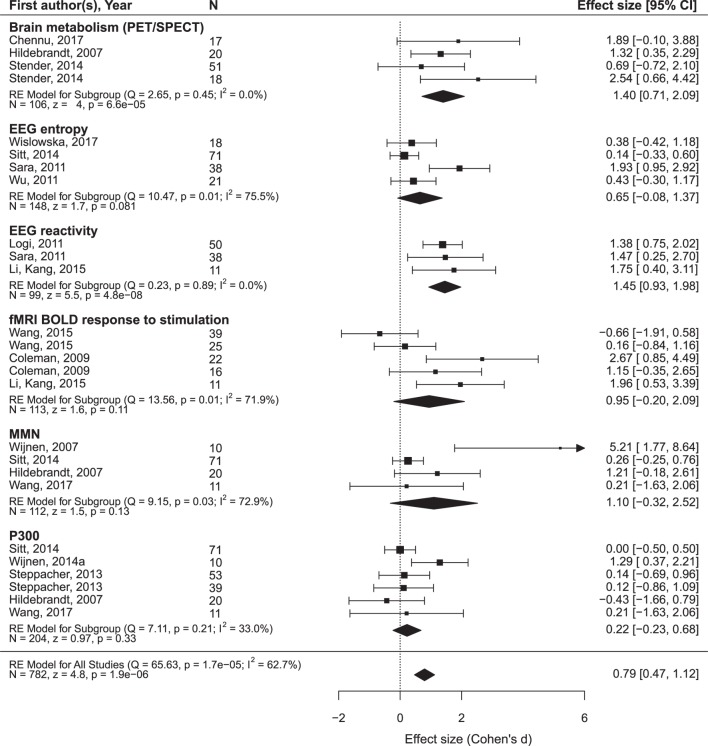
The results of the meta-analysis for prediction of the outcome from neurophysiological variables. The criterion of improvement for all patients was the recovery of consciousness. RE, random effects. The rest is the same as in Figure 3.

**Figure 6:**
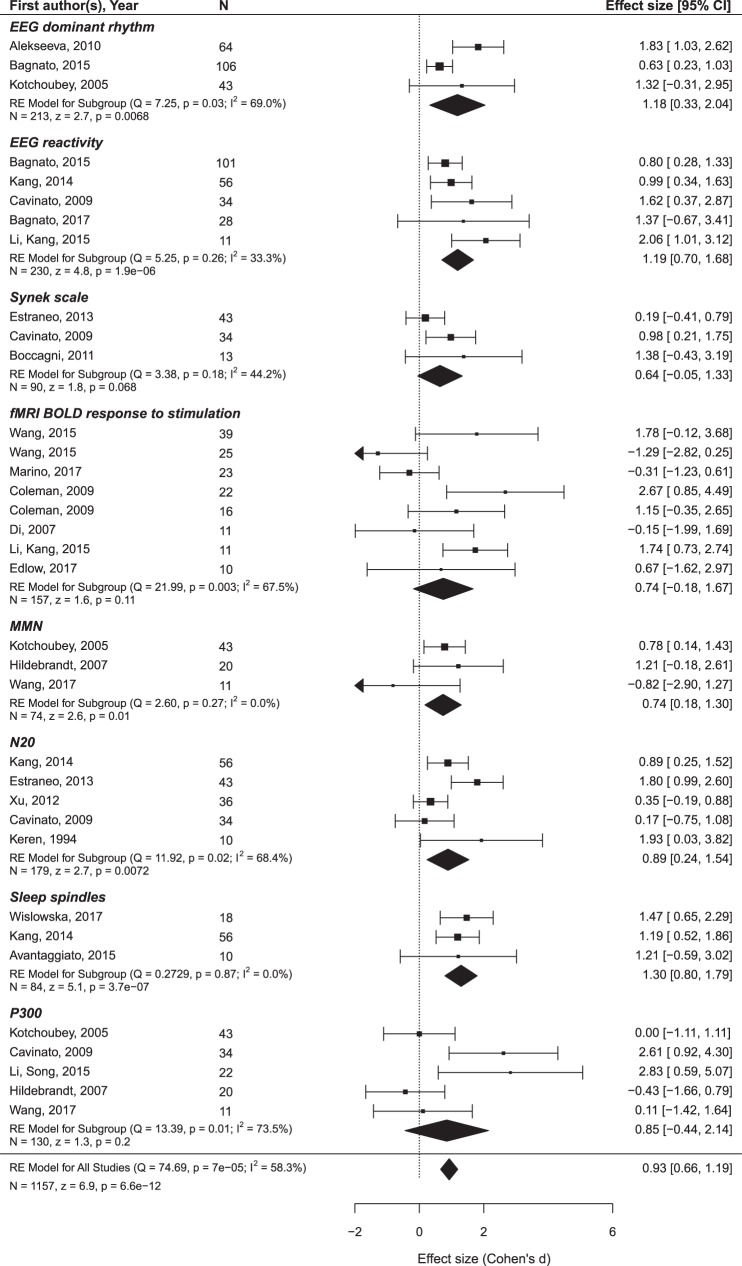
The results of the meta-analysis for prediction of the outcome from neurophysiological variables. The criterion of improvement for UWS patients was the transition to MCS, and for MCS patients, the recovery of consciousness. The rest is the same as in Figure 3.

The findings are summarized in Figures 5 and 6 and presented in more detail in Figures S1 and S2 and Tables S2 and S3 in Supplementary Material.

Only oscillatory EEG responses to passive stimulation appear to be reliably related to both prognostic criteria (i.e., minimal clinical improvement and the recovery of full consciousness). The included datasets are highly homogenous and yield a highly significant mean ES of 1.45 on a sample of 99 patients. Another prediction variable that significantly predicted the recovery of consciousness was brain metabolism assessed by PET/SPECT. It attained a mean ES of 1.40 on a sample of 106 patients. The prognostic value of the MMN, P300, EEG entropy variables, and fMRI responses to passive stimulation was not significant and characterized by strong heterogeneity of the primary datasets.^2^

More promising results have been obtained in relation to the minimal improvement criterion. In addition to the EEG reactivity, significant effects are found for the MMN and sleep spindles. Formally positive results are obtained for the SSEP component N20 and the background EEG frequency, but the data are too heterogeneous to make a conclusion.

## Discussion

Although the Section “Limitation” is frequently placed at the end of Discussion, we believe that particularly the discussion of meta-analytic data is useful to begin with limitations. One important limitation is that of the present work as such. As the manuscript was prepared for a special issue, we did not systematically address the authors of the original publications but relied solely on the published data including supplementary information. Although we believe that personal contact with the authors may have enhanced our knowledge, at the first step we did not use this strategy because it might have caused considerable delays.

Other limitations of the meta-analysis are rather related to the limitations of the primary literature. A quantitative review can overcome some limitations of the reviewed studies, e.g., their small size (thus, it can reveal a consistent and significant effect on the basis of several inconsistent and non-significant ones), but it cannot remove the biases implied in its empirical basis.

To begin with the least, the quality of data reporting is far from the present-day standard. While Fritz et al. (76) bristled that only 42% of empirical psychological studies report the size of their effects, in the present sample ES was mentioned only in five records (10.6%). Presenting a patient flow chart is already a standard in many fields of clinical research but fully unknown in the domain of DoC. About a half of the reviewed records neither describe inclusion and exclusion criteria nor even make a simple statement that all patients admitted to the hospital during some period were included. Thus, the concern of Kondziella et al. (7) about possible bias in patient selection seems to be justified.

The majority of the reviewed studies employed a univariate approach, i.e., each predictor was separately compared with the target variable. Of course, this is a serious limitation because we know that the values of a regression strongly depend on the other predictor variables included or excluded in/from the equation. Thus, a neurophysiological variable (e.g., P300), which appears useless as a single predictor, might reveal its effect in a particular combination with other predictors. A few groups have recently attempted to overcome this limitation and employed a multivariate approach to outcome prediction in DoC (24, 50). This seems to be a perspective line of research, but now the number of such records is still too low to undertake a separate meta-analysis of these data.

The negative correlation between the description of patient selection and the number of patients suggests that selection bias might be particularly strong in small-size studies. Although the sample size in prognostic studies is on average larger than in studies comparing UWS and MCS, we found, together with single case studies, 33 records with less than 20 patients, which implies that even in the case of the equal distribution at least one of the outcome groups (recovered or non-recovered) includes <10 patients. Particularly, the studies with the total *N* < 10 result in huge confidence intervals making any reliable conclusion impossible. Of course, small or even single case studies may have sense at the very beginning of the research process, when nothing is known about an investigated phenomenon whatsoever. However, in such a case, one can expect an increase of sample sizes with the year of publication, but this correlation was not significant.

Strong negative effects of underpowered studies on the quality of the reported data have thoroughly been discussed in the literature in general (63, 77, 78) and specifically in neuroscience (79, 80). Positive findings of tiny studies can only result from chance or selective data report. Our data show a consistent and significant correlation between the size of a prognostic effect and its standard error, indicating that stronger effects are less reliable. The correlation even withstood the removal of all datasets with <10 patients. As these small samples yield particularly large SE, the variability of SE was severely restricted, which might be expected to reduce the correlation coefficient. This was, however, not the case (Figure 2).

Another important limitation of nearly all studies was the lack of outcome blindness. Only two groups of authors clearly indicated that the diagnosis of the outcome was performed by neurologists without the knowledge of predictor values. One might argue that the diagnosis of recovery of consciousness (based on the criteria of consistent communication and functional use of objects) is quite easy and can hardly be biased by neurophysiological data. Even if this argument is true for exit-MCS, it is obviously false for the other broadly used improvement criterion of UWS patients, namely the transition to MCS. As the differential diagnostics between UWS and MCS is notoriously difficult (81), any information about positive or negative neurophysiological findings could influence the diagnostic decision. If this influence really takes place, we can expect much stronger correlation of neurophysiological indices with the transition to MCS than with the transition to exit-MCS, because the latter diagnosis is easier and thus less affected by additional information.

Exactly this was true. When the improvement criterion for both diagnostic groups (UWS and MCS) was the transition to exit-MCS, the weighted average ES (in terms of Cohen’s *d*) was rather moderate, in any case slightly smaller than 0.5. But the transition of UWS patients to MCS (which is quite difficult from the diagnostic point of view) was strongly related to the neurophysiological findings, with the weighted average ES being >1.4.

A potentially strong but still underestimated bias is related to unreported predictor variables. In most of the reviewed records, outcome prediction was not the main aim of the study, but rather a by-product of other analyses. Particularly, in such studies (though not only in these), many variables could be measured but not really reported. Sometimes, many variables are used in a UWS/MCS comparison but not even mentioned in relation to prognosis, although one may suppose that they were also compared with the follow-up data. Less frequently prognostic effects are referred to as “lacking” or “non-significant” without further quantification. Simmons et al. (65) suggest a very simple solution of the problem: whenever authors list their variables, they should add a short word “only.” We tried to counteract this false positive effect by assigning the value of 0 to the effects of obviously omitted variables (with its SE being assumed as the median SE of the reported variables). However, this method is not only imprecise but can also be biased, first, because the real number of such omitted variables may be much larger than a reader can guess, and second, because negative effects (i.e., those which run against the starting hypothesis, such as better neurophysiological responses in non-recovered patients) can be omitted more frequently than positive effects.

With this in mind, we understand that the data of meta-analyses should be taken with great caution. Nevertheless, we believe that a glance on the meta-analytic results can be of interest. First, the analysis was strongly complicated by the high variability of the primary records. Very small number of studies using exactly the same predictor and the same improvement criterion enforced us to combine similar methods, which resulted in high heterogeneity indices such as *I*^2^. Poor prognostic features of the characteristics of fMRI reactivity might partially be attributed to this group of studies including fMRI responses to very different stimulations from pain to music. For the same reason, we excluded some possible predictors (e.g., responses to active behavioral instructions; ERP N400 component) that were employed in two records only.

Second, the empirical contribution of predictors does not necessarily follow their general theoretical value defined by basic neuroscience. This is quite demonstrative in the case of P300, one of the most useful and most widely employed indices in neuroscience whose effect in the prediction of the outcome turned out to be virtually 0. One might speculate that P300 is not immediately related to consciousness (37), but, rather, to a more specific function such as working memory. Another possible reason may be the extreme difficulty of the separation between different P300 subcomponents (P3a and P3b) in the target population (38). The subcomponents are usually distinguished by topography and responses to active instruction, but most DoC patients have changed ERP topography and do not respond to instruction. If the results of this preliminary analysis should be used to determine which lines of research should not be recommended for future studies, P300 is the first candidate for such a negative recommendation. Also, the importance of the (highly expensive) fMRI predictors might similarly be overestimated on the basis of their theoretical importance.

Oscillatory EEG responses to stimulation showed, to the contrary, most promising effects, which agree well with the results of Bender et al. (10) obtained on the basis of different data.

Although EEG reactivity was measured to very different stimuli [e.g., to passive (23) or forced eyes opening (19, 20), to pain (34, 41), to warm water (39), and no description was given by Sarà et al. (48)] and the definition of reactivity substantially varied, the results are very homogenous across the reports. Moreover, this was the only group of predictor variables whose predictive value was significant for both improvement criteria (transition from UWS to MCS and the recovery of consciousness). Publication bias was also presented in these data, but it was less strong than for many other predictors (see Tables S2 and S3 in Supplementary Material).

Other perspective variables are brain metabolism (estimated by means of PET or SPECT) and the presence of sleep spindles in the EEG. Recent sleep data indicate a vital importance of spindles in information processing during sleep, which affects numerous cognitive processes in the subsequent wakefulness (75, 82, 83). DoC belongs to rare medical conditions characterized by severe deficits, or even complete absence, of sleep spindles, also in patients with relatively preserved sleep structure (84, 85). We believe that the role of sleep spindles in the outcome prediction in DoC should be explored in future work.

Both MMN and SSEP are proven outcome predictors for acute coma (86, 87). However, their value for the chronic DoC remains unclear. The present findings indicate their prognostic effects for the transition from UWS to MCS but not for recovery of consciousness.

The data further show that the predictive value of the auxiliary (e.g., neurophysiological) variables should be compared with the values of clinical variables. In the currently reviewed data, MCS patients had about 4.5 times better chances (if we take the lower limit of the 95% CI, three times better chances) to regain consciousness than UWS patients. It is true that we analyzed only records implementing the neurophysiological approach and missed similar data in the other publications not using neurophysiological variables. Nevertheless, the effect is very strong and, as far as we can judge, not strongly biased (as it was not a desired effect). This indicates the necessity to integrate neurophysiological and clinical predictors within a multivariate approach in further studies. This integration could be more productive in both diagnostic and prognostic respects than the attempts to oppose different classes of variables to each other.

These considerations can only be conceived of as preliminary. It should be stressed that all biasing factors discussed above act in the same direction, potentially increasing the number of *false positive* results. The general critique of Brok et al. (64) remains valid also for the current study: as long as the original studies do not present all information, meta-analyses can only try to diminish, but not abolish the positive bias. If we want to eliminate the bias, (i) small-size studies should be avoided (for prediction studies, groups of recovered/non-recovered should include at least 20 patients each); (ii) a flow chart should make evident the procedure of patient selection; (iii) neurologists assessing the target variable (i.e., change of the diagnosis) should be completely blinded regardless the values of neurophysiological predictors; (iv) the full list of measured variables including all potential predictors should be presented from the beginning of a report (in a Methods section); (v) the intervals (a) between the accident and the neurophysiological measurement, and (b) between this measurement and the follow-up assessment should be specified; finally, (vi) all positive *and negative* (e.g., non-significant relationships) results should be described in the same quantitative manner, either including the size of all effects, or permitting to calculate this size (e.g., mean and SD for the recovered and non-recovered groups).

Therefore, the numbers presented in Figures 5 and 6 and in the Supplementary Materials can now be regarded, not as estimates of real effects, but rather, as upper limits of these effects. The current state of affairs is yet far away from the level at which any practical recommendation can be given except the recommendation to be highly careful with interpretations.

## Author Contributions

Both authors performed data search and analyses. The manuscript was initiated by the second author and finalized by the first author.

## Conflict of Interest Statement

The authors declare that the research was conducted in the absence of any commercial or financial relationships that could be construed as a potential conflict of interest. The reviewer AP and handling Editor declared their shared affiliation.

## Footnotes

^1^Practically, “full consciousness” is defined as reliable communication or functional use of objects. In the following, when we speak about “regaining of consciousness” we shall mean these two abilities (at least one of them) and not just minimal signs of consciousness presented also in the MCS.

^2^The ES in Wijnen et al. (57) strongly differs from all other effects in the MMN group. After removing this result, the MMN data become completely homogenous (I2 = 0).
