# Supplementary material for: A Systematic Review and Meta-Analysis of the Relationship Between Brain Data and the Outcome in Disorders of Consciousness
Source: Front Neurol. 2018 May 8;9:315. doi: 10.3389/fneur.2018.00315 (PMC5954214; doi:10.3389/fneur.2018.00315)
Supplement: Supplementary file 1 [file Data_Sheet_1.DOCX]

**Supplementary Materials**

A systematic review and meta-analysis of the relationship between brain data and the outcome in Disorders of Consciousness

Boris Kotchoubey and Yuri G. Pavlov

**Supplementary Table 1 –** List of the records excluded from systematic review

| Study | Reason for exclusion |
| --- | --- |
| **Records containing no novel outcome data for DoC** | |
| Belkin, A. A., Alekseeva, E. V., Alasheev, A. M., Davydova, N. S., et al. (2017). Evaluation of circadence to predict the outcome of a vegetative state. *Consilium Medicum*, *19*(2), 19–23. | Data have already been published elsewhere |
| Boly, M., Garrido, M. I., Gosseries, O., Bruno, M.-A., et al. (2011). Preserved Feedforward But Impaired Top-Down Processes in the Vegetative State. *Science*, *332*(6031), 858–862. <https://doi.org/10.1126/science.1202043> | Lacking prognostic data |
| Faugeras, F., Rohaut, B., Weiss, N., Bekinschtein, T., et al. (2012). Event related potentials elicited by violations of auditory regularities in patients with impaired consciousness. *Neuropsychologia*, *50*(3), 403–418. | Lacking prognostic data |
| Molteni, E., Avantaggiato, P., Formica, F., Pastore, V., et al. (2016). Sleep/Wake Modulation of Polysomnographic Patterns has Prognostic Value in Pediatric Unresponsive Wakefulness Syndrome. *Journal of Clinical Sleep Medicine*, *12*, *12*(8, 8), 1131, 1131–1141. | Data have already been published elsewhere |
| Real, R. G. L., Veser, S., Erlbeck, H., Risetti, M., et al. (2016). Information processing in patients in vegetative and minimally conscious states. *Clinical Neurophysiology*, *127*(2), 1395–1402. | Lacking prognostic data |
| Risetti, M., Formisano, R., Toppi, J., Quitadamo, L. et al. (2013). On ERPs detection in disorders of consciousness rehabilitation. *Frontiers in Human Neuroscience*, *7*. <https://doi.org/10.3389/fnhum.2013.00775> | Lacking prognostic data |
| Zanatta, P., Messerotti Benvenuti, S., Baldanzi, F., & Bosco, E. (2012). Pain-related middle-latency somatosensory evoked potentials in the prognosis of post anoxic coma: a preliminary report. *Minerva Anestesiologica*, *78*(7), 749–756. | Patients in acute coma |
| **Records containing novel outcome data for DoC** | |
| Bagnato, S., Boccagni, C., Prestandrea, C., Sant’Angelo, A. et al. (2010). Prognostic value of standard EEG in traumatic and non-traumatic disorders of consciousness following coma. *Clinical Neurophysiology*, *121*(3), 274–280. | Data of MCS, UWS and conscious patients reported together |
| Bekinschtein, T., Tiberti, C., Niklison, J., Tamashiro, M., et al. (2005). Assessing level of consciousness and cognitive changes from vegetative state to full recovery. *Neuropsychological Rehabilitation*, *15*(3–4), 307–322. | Case study |
| de Jong, B. M., Willemsen, A. T. M., & Paans, A. M. J. (1997). Regional cerebral blood flow changes related to affective speech presentation in persistent vegetative state. *Clinical Neurology and Neurosurgery*, *99*(3), 213–216. | Case study |
| Faran, S., Vatine, J. J., Lazary, A., Ohry, A., et al. (2006). Late recovery from permanent traumatic vegetative state heralded by event-related potentials. *Journal of Neurology, Neurosurgery & Psychiatry*, *77*(8), 998–1000. | Case study |
| Fingelkurts, A. A., Fingelkurts, A. A., Bagnato, S., Boccagni, C., & Galardi, G. (2016). The Chief Role of Frontal Operational Module of the Brain Default Mode Network in the Potential Recovery of Consciousness from the Vegetative State: A Preliminary Comparison of Three Case Reports. *The Open Neuroimaging Journal*, *10*, 41–51. | Case study |
| Fingelkurts, A. A., Fingelkurts, A. A., Bagnato, S., Boccagni, C., & Galardi, G. (2011). Life or Death: Prognostic Value of a Resting EEG with Regards to Survival. *PLoS ONE*, 6, 25967. | The outcome measure was survived/died rather than improved/ unimproved |
| Fingelkurts, A. A., Fingelkurts, A. A., Bagnato, S., Boccagni, C., & Galardi, G. (2016). Long-Term (Six Years) Clinical Outcome Discrimination of Patients in the Vegetative State Could be Achieved Based on the Operational Architectonics EEG Analysis: A Pilot Feasibility Study. *The Open Neuroimaging Journal*, *10*, 69–79. | Case study |
| Giubilei, F., Formisano, R., Fiorini, M., Vitale, A., et al. (1995). Sleep abnormalities in traumatic apallic syndrome. *Journal of Neurology, Neurosurgery & Psychiatry*, *58*(4), 484–486. | Neither individual patients’ data nor essential statistics are reported |
| Gnezditskiĭ, V. V., Popova, L. M., Fedin, P. A., Avdiunina, I. A., et al. (1996). Prognostic significance of neurophysiological parameters in nontraumatic apallic syndrome. *Anesteziologiia I Reanimatologiia*, *2*, 16–21. | No full text available |
| Golkowski, D., Merz, K., Mlynarcik, C., Kiel, T., Schorr, B., Lopez-Rolon, A., … Ilg, R. (2017). Simultaneous EEG–PET–fMRI measurements in disorders of consciousness: an exploratory study on diagnosis and prognosis. *Journal of Neurology*, *264*(9), 1986–1995. <https://doi.org/10.1007/s00415-017-8591-z> | Neither individual patients’ data nor essential statistics are reported |
| Gosseries, O., Schnakers, C., Ledoux, D., Vanhaudenhuyse, A., Bruno, M.-A., Demertzi, A., … Laureys, S. (2011). Automated EEG entropy measurements in coma, vegetative state/unresponsive wakefulness syndrome and minimally conscious state. *Functional Neurology, 26*(1), 25–30. | Data of coma and UWS reported together |
| Meiron, O., & Jaul, E. (2017). Paroxysmal theta power reactivity is related to survival in anoxic vegetative state patients. *Clinical Neurophysiology*, *128*(7), 1255–1257. | The outcome measure was survived/died rather than improved/ unimproved |
| Menon, D. K., Owen, A. M., Boniface, S. J., & Pickard, J. D. (1998). Cortical processing in persistent vegetative state. *The Lancet*, *352*(9134), 1148–1149. | Case study |
| Moritz, C. H., Rowley, H. A., Haughton, V. M., Swartz, K. R., et al. (2001). Functional MR imaging assessment of a non-responsive brain injured patient. *Magnetic Resonance Imaging*, *19*(8), 1129–1132. | Case study |
| Owen, A. M., Coleman, M. R., Boly, M., Davis, M. H., et al. (2006). Detecting Awareness in the Vegetative State. *Science*, *313*(5792), 1402–1402. <https://doi.org/10.1126/science.1130197> | Case study |
| Owen, A. M., Coleman, M. R., Menon, D. K., Johnsrude, I. S., et al. (2005). Residual auditory function in persistent vegetative state: a combined PET and fMRI study. *Neuropsychological Rehabilitation*, *15*(3–4), 290–306. | Case study |
| Owen, A. M., Menon, D. K., Johnsrude, I. S., Bor, D., Scott, S. K., Manly, T., Pickard, J. D. (2002). Detecting residual cognitive function in Persistent Vegetative State. *Neurocase*, *8*(5), 394–403. | Case study |
| Schnakers, C., Ledoux, D., Majerus, S., Damas, P., et al. (2008). Diagnostic and prognostic use of bispectral index in coma, vegetative state and related disorders. *Brain Injury*, *22*(12), 926–931. | Data of coma and UWS reported together |
| Sharova, E. (2014). *EEG Correlates of Consciousness Recovery after Traumatic Brain Injury* (Vol. 1). | Neither individual patients’ data nor essential statistics are reported |
| Staffen, W., Kronbichler, M., Aichhorn, M., Mair, A., & Ladurner, G. (2006). Selective brain activity in response to one’s own name in the persistent vegetative state. *Journal of Neurology, Neurosurgery & Psychiatry*, *77*(12), 1383–1384. <https://doi.org/10.1136/jnnp.2006.095166> | Case study |
| van den Brink, R. L., Nieuwenhuis, S., van Boxtel, G. J. M., van Luijtelaar, G., et al. (2018). Task-free spectral EEG dynamics track and predict patient recovery from severe acquired brain injury. *NeuroImage: Clinical*, *17*(Supplement C), 43–52. <https://doi.org/10.1016/j.nicl.2017.10.003> | Neither individual patients’ data nor essential statistics are reported |
| Wu, X., Zou, Q., Hu, J., Tang, W., et al. (2015). Intrinsic Functional Connectivity Patterns Predict Consciousness Level and Recovery Outcome in Acquired Brain Injury. *Journal of Neuroscience*, *35*(37), 12932–12946. <https://doi.org/10.1523/JNEUROSCI.0415-15.2015> | Neither individual patients’ data nor essential statistics are reported |

***Supplementary Figure 1*** – Funnel plots outcome with potentially missing studies detected by “trim-and-fill” technique (represented by empty circles; see Duval & Tweedie, 2000; Peters et al., 2007) for each predictor group. Improvement criterion for UWS is transition to MCS or better

***Supplementary Figure 2*** – Funnel plots outcome with potentially missing studies detected by “trim-and-fill” technique (represented by empty circles; see Duval & Tweedie, 2000; Peters et al., 2007) for each predictor group.

***Supplementary Figure 3*** – Funnel plots for the outcomes based only on the diagnosis with potentially missing studies detected by “trim-and-fill” technique (represented by empty circles; see Duval & Tweedie, 2000; Peters et al., 2007). Improvement criterion for UWS is regaining of consciousness (left panel) or transition to MCS (right panel).

**Supplementary Table 2** – Publication bias statistics for the “minimal improvement” criterion

| Predictor | Corrected for bias  Effect size  [-CI +CI] | Rosenthal File Drawer Analysis | | Regression test for Funnel plot asymmetry (Egger's test) | |
| --- | --- | --- | --- | --- | --- |
|  |  | Fail-safe N | Observed p | z | p |
| ***P300*** | 0.50 [-0.82 1.82] | 5 | 0.0134 | 2.817 | 0.005 |
| ***Sleep spindles*** | 1.29 [0.79 1.79] | 23 | <.0001 | 0.033 | 0.974 |
| ***N20*** | 0.79 [0.18 1.40] | 38 | <.0001 | 1.009 | 0.313 |
| ***MMN*** | 0.74 [0.18 1.29] | 2 | 0.0273 | -0.809 | 0.419 |
| ***fMRI to stim.*** | 0.74 [-0.18 1.69] | 15 | 0.0014 | 0.395 | 0.693 |
| ***Synek scale*** | 0.18 [-0.56 0.94] | 5 | 0.0040 | 1.097 | 0.273 |
| ***Reactivity*** | 0.92 [0.36 1.49] | 65 | <.0001 | 1.663 | 0.096 |
| ***Background*** | 0.63 [-0.27 1.53] | 29 | <.0001 | 0.564 | 0.573 |
| *All studies* | 0.84 [0.57 1.12] | 1409 | <.0001 | 0.973 | 0.330 |

**Note:** This quantitative assessment of publication bias for each group of neurophysiological predictor variables complements the graphical assessment presented in Supplementary Figure 1. Rosenthal test (Rosenthal, 1979) indicates how many studies yielding a null result would have to remain in the file drawer if we suppose that in reality the null hypothesis is true. The higher this number, the more resistant to the file drawer threat is the empirical result that rejects the null hypothesis. Egger’s test (Egger et al., 1997) is similar to the correlative investigation of publication bias presented in the main text but uses regression analysis rather than correlation analysis. The test presumes that in the absence of publication bias, the regression line of the standard normal deviate (defined as effect size divided by its SE) on the precision (defined as 1/SE) should have an intercept of zero. The larger the deviation of the intercept from zero, the higher the risk of publication bias.

**Supplementary Table 3** – Publication bias statistics for the “regaining consciousness” improvement criterion

| Predictor | Corrected for bias  Effect size  [-CI +CI] | Rosenthal File Drawer Analysis | | Regression test for Funnel plot asymmetry (Egger's test) | |
| --- | --- | --- | --- | --- | --- |
|  |  | Fail-safe N | Observed p | z | p |
| ***P300*** | 0.2246 [-0.23 0.68] | 0 | 0.1229 | 0.022 | 0.982 |
| ***MMN*** | 1.09 [-0.32 2.52] | 9 | 0.0016 | 9.146 | 0.027 |
| ***fMRI to stim.*** | 0.16 [-1.16 1.49] | 10 | 0.0023 | 2.127 | 0.033 |
| ***Reactivity*** | 1.38 [0.93 1.84] | 28 | <.0001 | 0.413 | 0.679 |
| ***Entropy*** | 0.65 [-0.08 1.37] | 12 | 0.0006 | 2.02 | 0.043 |
| ***PET/SPECT*** | 1.11 [0.50 1.73] | 21 | <.0001 | 0.871 | 0.384 |
| *All studies* | 0.53 [0.17 0.90] | 533 | <.0001 | 3.216 | 0.001 |

**Note:** This quantitative assessment of publication bias for each group of neurophysiological predictor variables complements the graphical assessment presented in Supplementary Figure 2. Else the same as in Supplementary Table 2.

**Supplementary Table 4** – Publication bias statistics for the outcomes based only on diagnosis

| Improvement criteria for UWS | Corrected for bias  Effect size  [-CI +CI] | Rosenthal File Drawer Analysis | | Regression test for Funnel plot asymmetry ("Egger's test") | |
| --- | --- | --- | --- | --- | --- |
|  |  | Fail-safe N | Observed p | z | p |
| ***Regaining consciousness*** | 0.944 [0.72 1.17] | 199 | <0.0001 | -1.2532 | 0.2101 |
| **at least MCS** | 0.04 [-0.26 0.34] | 0 | 0.4909 | -0.6653 | 0.5059 |

**Supplementary References**

Duval, S., & Tweedie, R. (2000). Trim and fill: A simple funnel-plot-based method of testing and adjusting for publication bias in meta-analysis. Biometrics, 56, 455-463.

Egger, M., Smith, G. D., Schneider, M., & Minder, C. (1997). Bias in meta-analysis detected by a simple, graphical test. British Medical Journal, 315, 629.

Peters, J. L., Sutton, A. J., Jones, D. R., Abrams, K. R., & Rushton, L. (2007). Performance of the trim and fill method in the presence of publication bias and between-study heterogeneity. Statistics in Medicine, 26, 4544-4562.

Rosenthal, R. (1979). The "file drawer problem" and tolerance for null results. Psychological Bulletin, 86(3), 638-641.
